# Respiratory Muscle Function in Children and Adolescents with Cystic Fibrosis in the Era of CFTR Modulator Therapies

**DOI:** 10.3390/children12070878

**Published:** 2025-07-03

**Authors:** Guillermo García-Pérez-de-Sevilla, Ángela Blanco Velasco, Thomas Yvert, Verónica Sanz-Santiago, Ana Morales Tirado, Alejandro López Neyra, Cristina de Manuel, Marta Ruiz Valbuena, Margarita Pérez-Ruiz

**Affiliations:** 1 Department of Physiotherapy, Faculty of Medicine, Health and Sports, European University of Madrid, 28670 Madrid, Spain; 2 ImFINE Research Group, Department of Health and Human Performance, Facultad de Ciencias de la Actividad Física y del Deporte, INEF Universidad Politécnica de Madrid, 28040 Madrid, Spainthomas.yvert@upm.es (T.Y.); margarita.perez@upm.es (M.P.-R.); 3 Cystic Fibrosis Unit, Pediatric Pulmonology Department, Hospital Universitario Infantil Niño Jesús de Madrid, 28009 Madrid, Spain; vsanzs@salud.madrid.org (V.S.-S.); alneyra@salud.madrid.org (A.L.N.); 4 Cystic Fibrosis Unit, Department of Pediatric Medicine, Faculty of Medicine and Health Sciences, Ramon y Cajal University Hospital, 28034 Madrid, Spain; ana.moralest@salud.madrid.org; 5 Pediatric Pulmonology Department and Cystic Fibrosis Unit, Hospital La Paz, 28046 Madrid, Spain; cristina.demanuel@salud.madrid.org (C.d.M.); marta.ruizdevalbuena@salud.madrid.org (M.R.V.)

**Keywords:** cystic fibrosis, CFTR modulators, maximal inspiratory pressure, maximal expiratory pressure

## Abstract

**Objective**: The objective of this study was to analyze respiratory muscle function in children and adolescents with cystic fibrosis (CF) treated with Elexacaftor/Tezacaftor/Ivacaftor (ETI) compared to healthy individuals, based on the hypothesis that CFTR modulators may improve respiratory muscle strength. **Methods**: A descriptive, observational, cross-sectional study was conducted with patients with CF treated with ETI aged 6–18 years. Lung function, maximal expiratory and inspiratory pressures (MIP and MEP), diet quality (KIDMED), and physical activity levels (PAQ) were assessed. The student’s *t*-test or the Mann–Whitney U-test was used to compare differences between groups. The effect size was calculated with Cohen’s d. Significance level was set as a *p*-value < 0.05. **Results**: A total of 48 children and adolescents (60.4% male) were analyzed in this study (24 healthy and 24 with CF). The participants with CF had mild pulmonary involvement. No significant differences were found in respiratory muscle strength between groups (MEP_max_ *p* = 0.440, MIP_max_ *p* = 0.206). Patients with CF showed lower KIDMED (*p* = 0.022) and PAQ (*p* = 0.010) scores. However, the MIP and MEP values observed in CF participants were higher than those reported in previous studies conducted before the introduction of ETI modulators. **Conclusions**: Children and adolescents with CF treated with ETI showed respiratory muscle strength comparable to that of healthy controls. Despite differences in lifestyle factors, these findings may reflect a positive impact of CFTR modulators on respiratory muscle function, although further longitudinal and controlled studies are needed.

## 1. Introduction

Cystic fibrosis (CF) is a complex, inherited, autosomal recessive disorder that primarily affects the respiratory, digestive, and reproductive systems [1]. It is caused by mutations in the cystic fibrosis transmembrane conductance regulator (CFTR) gene, which results in defective chloride ion transport, leading to thick, sticky mucus accumulation. This mucus impairs the normal functioning of organs, especially the lungs, and results in chronic respiratory infections, inflammation, and progressive lung damage. CF is one of the most common life-limiting genetic disorders, with an estimated 80,000 individuals affected globally, according to recent reports from both the European Cystic Fibrosis Society and the US Cystic Fibrosis Foundation [2,3].

The clinical manifestations of CF are highly variable, with the severity of disease progression being influenced by several factors, including genetic mutations, age of diagnosis, and environmental exposures. However, one of the most significant determinants of clinical outcomes in CF is the individual’s lifestyle, particularly their engagement in physical exercise and adherence to healthy nutritional patterns, such as the Mediterranean Diet [4]. Regular physical activity and adequate nutrition are essential components of CF care, as they support airway clearance, respiratory muscle strength, and overall quality of life [5,6].

Despite the benefits of a healthy lifestyle, CF remains a progressive disease, and the natural deterioration of lung function remains inevitable. In children and adolescents, lung involvement is often present from early childhood, with chronic respiratory symptoms, such as cough, wheezing, and recurrent infections, often exacerbated by the thickened mucus that obstructs the airways [7,8]. Over time, the continuous cycle of inflammation and infection leads to irreversible lung damage, manifesting as bronchiectasis, fibrosis, and progressive decline in pulmonary function. This clinical course underscores the need for continuous monitoring and early intervention to prevent further deterioration. Recent advancements in CF care, including early diagnosis and novel pharmacological therapies, have modified the clinical trajectory of the disease for many patients [9,10,11].

Among the challenges faced by individuals with CF are altered breathing mechanics and reduced respiratory muscle strength. Maximum inspiratory pressure (MIP) and maximum expiratory pressure (MEP) are widely used measures of respiratory muscle function. Decreased MIP and MEP are indicative of respiratory muscle weakness, which can contribute to reduced ventilatory efficiency and decreased exercise tolerance, having as its main consequence the increase in dyspnea and CO2 retention before, during, and after exercise [6,12]. Several studies have demonstrated that children and adolescents with CF with moderate pulmonary involvement exhibit significantly lower MIP and MEP values compared to healthy peers, highlighting the impact of the disease on respiratory muscle performance [13,14,15].

These impairments in respiratory muscle strength can also affect other aspects of pulmonary function, including the ability to perform everyday physical tasks, which further contributes to the overall burden of the disease. Notably, although respiratory muscle strength is generally lower in CF patients, the relationship between MEP, MIP, and overall lung function can be complex. While a decrease in MEP is often correlated with poor lung function in these patients, MIP does not always follow this pattern, indicating the multi-faceted nature of respiratory dysfunction in CF [16,17]. With advancing age, patients may rely more on accessory muscles, worsening breathing efficiency, and exertional dyspnea [18]. Inspiratory muscle strength could be a key factor in evaluating the impact of pulmonary rehabilitation in this specific disease [12].

With the advent of CFTR modulators, important advances have been made in the treatment of CF. These drugs, such as Ivacaftor, Lumacaftor/Ivacaftor, and the more recent triple-combination therapies like Elexacaftor/Tezacaftor/Ivacaftor (ETI), have revolutionized CF care by targeting the underlying defect in the CFTR protein. ETI, for instance, has been shown to improve lung function, reduce pulmonary exacerbations, and enhance quality of life, particularly in patients with the most common CFTR mutation, F508del [19,20,21]. Nonetheless, the full spectrum of effects—particularly on respiratory muscle strength—is still under investigation [22].

In patients treated with ETI, one might expect an improvement in both MIP and MEP values, aligning more closely with those observed in healthy children and adolescents. If these medications can successfully restore normal CFTR function, they may help mitigate the compensatory respiratory muscle weakness seen in CF patients, potentially normalizing pressures such as MIP and MEP [23]. However, this remains speculative, as the full effects of these therapies on respiratory mechanics are still being explored in clinical settings. In the long term, it is hoped that ETI will not only preserve lung function but also may be associated with improved functional impairments that have traditionally been seen in CF patients, such as MIP and MEP [24,25].

Ultimately, as CF care continues to evolve with the advent of novel therapies, it is crucial to re-evaluate how these treatments influence not just pulmonary function but also the associated respiratory muscle strength and overall functional capacity in children and adolescents with CF. These insights will guide future therapeutic strategies and provide a more comprehensive understanding of the disease trajectory in the modern era of CF treatment. The assessment of respiratory muscle strength is a simple and informative clinical tool in this context. Therefore, the objective of this study was to analyze respiratory muscle function in children and adolescents with CF treated with ETI compared to healthy individuals. This study tests the null hypothesis that in children and adolescents with mild pulmonary involvement and limited disease progression, there should not be significant differences in respiratory muscle function.

## 2. Methods

### 2.1. Study Design and Ethical Considerations

A descriptive, observational, cross-sectional study was conducted in accordance with the Declaration of Helsinki. This study was approved by the Ethical Research Committee of the Hospital Universitario Niño Jesús, Madrid (SPAIN), with the registration number R-0011/23. All participants and guardians signed an informed consent form to participate in this study. In addition, the Strengthening the Reporting of Observational Studies in Epidemiology (STROBE) [26] statement was used as a reference for writing the manuscript.

### 2.2. Participants

Healthy participants (n = 24) and participants diagnosed with CF with ETI treatment (n = 24) aged 6–18 years were included in the present study. This sample was recruited by convenience sampling. The CF patients were recruited by convenience sampling from three hospitals in the Community of Madrid, while healthy controls were recruited from a local school near the hospitals in the same area of the city. The sample was screened according to the selection criteria for this study. The following eligibility criteria for the patients with CF were used: (a) children and adolescents aged 6–18 years; (b) diagnosed with CF pathology with at least 6 months of ETI treatment; (c) clinically stable at the time of assessment; (d) available for static and dynamic lung function tests. Healthy controls were children and adolescents aged 6–18 years without pulmonary disease or infection.

While exclusion criteria were (a) participants with symptoms associated with a pulmonary exacerbation during the last month; (b) patients diagnosed with or in the presence of another respiratory or cardiac disease linked to symptoms of persistent respiratory dysfunction or during exercise; (c) locomotor difficulties affecting the assessment protocol; (d) inadequate use of medication prescribed by the medical service; (e) patients with an FEV1 < 60%, to avoid severe disease confounding. In addition, no medication was withdrawn during the test days, and patients maintained their usual treatment regimen.

Due to the limited availability of pediatric CF patients meeting these criteria, the sample size was constrained. Controls were matched to CF patients on a one-to-one basis by age and sex.

Finally, a pairwise comparison was made between groups (CF and healthy) by sex, age, weight, and height.

### 2.3. Variables

Assessments of the participants were performed within a multidisciplinary team at the hospital consisting of specialist physicians, pediatricians, nutritionists, nurses, physiotherapists, and sports scientists. The variables measured were lung function (only in CF patients), MEP, MIP, adherence to the Mediterranean diet, and physical activity (PA). Also, demographic variables such as age, weight with a mechanical scale (Asimed^®^ Barys Plus C, Barcelona, Spain), and height with a telescopic measuring rod were measured. Then, Body Mass Index (BMI, kg/m^2^) was calculated.

#### 2.3.1. Lung Function

Spirometry was performed in which respiratory flows and volumes were assessed based on the following variables: forced vital capacity (FVC); forced expiratory volume in the first second (FEV1); FEV1/FVC ratio; and forced expiratory flow between 25 and 75% of forced vital capacity (FEF25–75%). Spirometry was performed using a Master Screenspirometer (Jaeger^®^ Vyntus PNEUMO, Höchberg, Germany) following the American Thoracic Society-European Respiratory Society (ATS/ERS) guideline 1. The data were interpreted using the unified approach of the Global Lung Initiative (GLI) of 2012 [27,28,29].

#### 2.3.2. Maximal Expiratory and Inspiratory Pressures

MIP and MEP (cm H_2_O) were measured using a handheld respiratory manometer (MicroRPM^®^, CareFusion, Hoechberg, Germany) and following the American Thoracic Society/European Respiratory Society recommendations [30]. The participants rested for five minutes before performing the first maneuver. Then, they performed the maneuvers in a seated position, with their back straight, holding their nose to prevent air leaks. The examiner previously showed the maneuver and assessed its correct execution.

Participants started with the MEP measurement: they were asked to inhale at the maximum inspiratory volume with a second in inspiratory apnea and then exhale as hard as they could in the device, keeping the lips firmly closed around it to prevent air leakage. Participants rested for one minute and repeated the maneuver three times. The maximum value of the three repetitions was taken.

Then, after 1 min of rest, the MIP measurement was performed: Participants were asked to exhale until the lungs were empty, hold for a second at maximum exhalation, and inhale as hard as possible. Participants rested for one minute and repeated the maneuver three times. The maximum value of the three repetitions was taken.

According to a recent systematic review about reference values for MIP and MEP in healthy children and adolescents, values from females 4–11 years were: 65.8 cmH_2_O for MIP and 72.8 cmH_2_O for MEP, and for males, 75.4 cmH_2_O for MIP and 84.0 cmH_2_O for MEP. In the 12–19 age group, values for females were 82.1 cmH_2_O for MIP and 90.0 cmH_2_O for MEP, and for males, they were 95.0 cmH_2_O for MIP and 105.7 cmH_2_O for MEP [31].

In addition, the MEP/MIP ratio was calculated, which is a valuable measure of diaphragm function. In previous studies, the mean MEP/MIP ratio values for children and adolescents are near 1.0 [32,33].

#### 2.3.3. Diet Quality

Diet quality was assessed using the validated Mediterranean Diet Quality Index (KIDMED) questionnaire [34], which measures adherence to the Mediterranean diet in children and adolescents. It consists of 16 yes/no questions with an assigned score. According to the score obtained, the quality of the diets is classified into three categories: poor quality diet (≤3 points), need to improve the dietary pattern (from 4 to 7 points), and optimal Mediterranean diet (≥8 points).

#### 2.3.4. Physical Activity

The Physical Activity Questionnaire for Children (PAQ-C; ages 8–12 years) [35] and Adolescents (PAQ-A; ages 13–18 years) [36] was used to ascertain the PA level of the patients. It is a questionnaire that assesses PA levels in children and adolescents with a general estimate during school and after school in the last 7 days. The questionnaire consists of 10 questions in the PAQ-C and 9 in the PAQ-A, and the last question of both questionnaires allows us to know if the child or adolescent was ill or had a problem that prevented him/her from doing PA. The questions assess different aspects of PA using a 5-point Likert scale, and a score will be obtained from the average of 9 questions in the PAQ-C and 8 in the PAC-A. Thus, values < 2.5 mean that they do not meet the minimum PA recommendations.

### 2.4. Statistical Analysis

For the statistical analysis, descriptive and inferential statistical tests were used using IBM SPSS Statistics (SPSS) v 29.0 software. The mean and standard deviation were calculated for each of the items analyzed for the description of parametric variables. In addition, the normality of the data was checked using the Shapiro-Wilk test with *p* > 0.05. Then, to compare means between healthy and CF, the Student’s *t*-test was used for parametric variables and the Mann–Whitney U-test for non-parametric variables. The effect size was calculated with Cohen’s d, establishing values between 0.2 and 0.3 as a small effect size; a medium effect around 0.5–0.8; and a large effect from 0.8 onwards. Significance level was set as a *p*-value < 0.05.

## 3. Results

A total of 48 children and adolescents were analyzed in this study (24 healthy and 24 CF), with a predominance of boys (60.4%) over girls (39.6%).

### 3.1. Characteristics of the Sample

Descriptive results of the participants are shown in Table 1. Regarding weight, height, and age data, there were no significant differences between groups. In terms of lung function, the participants with CF had FEV_1_ values ≥ 80%, showing mild pulmonary involvement (Table 2).

### 3.2. MIP and MEP Results

The results shown in Table 3 show that there were no significant differences between the MEP and MIP assessments between the two populations (MEP_max_ *p* = 0.440, MIP_max_ *p* = 0.206). As for the results obtained in the KIDMED questionnaire, there were significant differences, with CF patients showing a lower score (*p* = 0.022), with a medium effect size. In addition, significant differences were also found in the PAQ values (*p* = 0.010), with CF patients showing lower PA values, with a medium effect size.

## 4. Discussion

Respiratory rehabilitation has been fundamental in managing this chronic pediatric respiratory condition. In this new era, where CFTR modulator therapies have significantly improved pulmonary function in patients with CF [37], examining variables such as respiratory muscle strength is pertinent. This study analyzed whether respiratory muscle strength, measured via MIP and MEP, approaches the levels seen in healthy children of the same age and sex. The healthy control group exhibited better physical activity levels and dietary quality compared to the CF group treated with ETI. Despite these lifestyle differences, no statistically significant differences were found between the two groups in MIP or MEP.

This finding is particularly relevant because it suggests the hypothesis that CFTR modulators may preserve respiratory muscle function that has historically been observed in pediatric CF populations. Prior studies in the pre-ETI era consistently demonstrated decreased MIP and MEP values in CF patients with comparable FEV1 levels (70–80%), often attributing this to muscle atrophy, chronic inflammation, and compromised energy metabolism [38,39].

Patients with CF often exhibit reduced muscle mass and strength in skeletal muscles, including the diaphragm, the primary muscle of respiration. CFTR deficiency intrinsically contributes to skeletal muscle atrophy and dysfunction [40]. In normal skeletal muscle of mice and humans, CFTR is expressed and localized alongside sarcoplasmic reticulum-associated proteins. CFTR-deficient myotubes show elevated intracellular calcium levels, which are essential in excitation-contraction coupling. Wells et al. demonstrated that CF patients have abnormal muscle bioenergetics both at rest and during exercise, characterized by lower intramuscular ATP levels, a reduced ATP/phosphocreatine (PCr) ratio at rest, and decreased PCr recovery rates post-exercise. This suggests altered aerobic metabolism in CF patients [41]. Additionally, the absence of CFTR in the diaphragm and limb myotubes is associated with a hyperinflammatory phenotype, including overexpression of pro-inflammatory cytokines and key regulators of the ubiquitin-proteasome system, linked to muscle mass loss. This exaggerated upregulation of cytokines, including tumor necrosis factor (TNF), during pulmonary infection further impairs diaphragmatic function [42,43]. Therefore, CFTR deficiency is a direct cause of diaphragmatic muscle dysfunction, potentially impacting MIP and MEP [44].

Prior to the ETI era, respiratory physiotherapy primarily aimed to improve mucus clearance and strengthen respiratory muscles [45]. Strengthening expiratory muscles through patient-specific rehabilitation protocols improved exercise tolerance [6]. Regarding respiratory muscle function, CF patients often exhibit lower-than-expected MIP and MEP values for their age, leading to increased dyspnea and CO2 levels before, during, and after exercise. Adequate strength and endurance of respiratory muscles are essential for optimal ventilatory response [46,47,48,49,50].

In this study, the absence of significant group differences in MIP, MEP, or MEP/MIP ratio, even in the context of suboptimal lifestyle parameters in the CF group, indicates a possible disease-modifying effect of ETI therapy on respiratory muscle integrity [23,24,25].

It is interesting that MIP and MEP values in our CF group were not only comparable to those of healthy controls but were also higher than those reported in pre-ETI studies involving children and adolescents with similar FEV1 scores [31]. This may suggest a potential positive effect of ETI on neuromuscular respiratory performance. Furthermore, this finding opens the possibility that some of the improvements in perceived exertion, exercise tolerance, and ventilatory efficiency observed clinically in ETI-treated patients may stem from improvements in respiratory muscle capacity, not solely from enhanced airway clearance and lung mechanics.

### 4.1. Clinical Implications

The clinical relevance of these findings lies in the fact that children with CF treated with ETI showed a respiratory muscle function comparable to their healthy peers—something that was rarely observed prior to the availability of CFTR modulators. This may have implications for how clinicians monitor and manage CF patients in the context of modern therapies.

Several studies have identified positive correlations between MIP and inspiratory muscle activation [51], MEP and expiratory muscle activation [52], aerobic capacity [46], and abdominal muscle mass [53]. These variables are critical determinants of clinical outcomes in pediatric CF, particularly during periods of infection or physical stress.

Currently, inspiratory and expiratory muscle strength assessments are not routinely performed in clinical respiratory consultations. However, given their simplicity, non-invasiveness, and high prognostic value, they represent an untapped opportunity in clinical practice. In this new era of CF care, with rapid therapeutic advances, it is essential to implement broader, more holistic assessment strategies that include respiratory muscle function to fully capture treatment benefits.

Moreover, systematic measurement of MIP and MEP may assist in tailoring respiratory physiotherapy programs. By identifying patients who still exhibit suboptimal muscle performance despite pharmacologic gains, interventions can be more effectively targeted and personalized. This may be particularly important in children with sedentary lifestyles or poor nutritional status, where ETI’s benefits might not fully translate into functional outcomes without adjunct rehabilitation strategies.

In summary, these findings support a broader view of how therapeutic success in CF may be assessed. Beyond spirometry, evaluating respiratory muscle strength offers additional insight into functional recovery and may help optimize long-term outcomes through individualized care strategies.

To our knowledge, this is the first study to directly compare MIP and MEP in ETI-treated pediatric CF patients versus healthy controls.

### 4.2. Limitations and Future Research Lines

This observational study cannot establish causality. Additionally, the sample was recruited by convenience sampling without prior sample size calculation. While we were able to access baseline lung function data (spirometry) prior to ETI initiation, respiratory muscle strength measures (MIP and MEP) were not assessed before treatment, which limits the ability to evaluate longitudinal changes in these variables. Moreover, a comparison group of CF patients not receiving ETI was not included. This was due to ethical and practical constraints, as withholding or delaying ETI in eligible patients is not justifiable in real-world clinical practice, given the substantial and rapid benefits of the therapy. Another limitation is that spirometric values were not collected in the healthy control group, under the assumption of normal lung function in asymptomatic individuals. However, this limited our ability to directly compare pulmonary function between groups.

## 5. Conclusions

This study found no significant differences between groups in MIP, MEP, or the MEP/MIP ratio. The groups were age- and sex-matched, with the only notable difference being that the healthy group had better physical activity levels and diet quality than the CF group treated with CFTR modulators. These results suggest that, in children and adolescents with CF and ETI treatment, with mild pulmonary involvement and limited disease progression, respiratory muscle function remains preserved and similar to healthy controls.

## Figures and Tables

**Table 1:** Descriptive data of the sample.

	HEALTHY (n = 24)	CF (n = 24)	*p* Value	Cohen’s d
**Demographics**				
Age (mean ± SD)	12.03 ± 3.18	12.51 ± 2.76	0.572	−0.164
Sex, men (n (%))	14 (57.3 %)	15 (62.5 %)	1.000	
**Anthropometrics**				
Height (cm)	147.54 ± 15.63	148.62 ± 12.89	0.795	−0.075
Height (z-score)	0.06 ± 0.99	−0.28 ± 0.80	0.202	0.372
Weight (kg)	41.63 ± 13.05	41.63 ± 12.78	0.998	−0.001
Weight (z-score)	0.06 ± 0.63	−0.32 ± 1.03	0.127	0.453
BMI (kg/m^2^)	18.56 ± 2.44	18.46 ± 3.62	0.913	0.032
BMI z score	0.15 ± 0.60	−0.27 ± 1.03	0.317	0.242

Note: Values expressed as mean ± SD, standard deviation, and with n, number, and %, percentage. Abbreviations: CF, cystic fibrosis; BMI, body mass index.

**Table 2:** Lung function in CF patients.

Lung Function	
FEV_1_ (L)	2.51 ± 0.71
FEV_1_ (%)	101.19 ± 14.03
FEV_1_ z-score	0.11 ± 1.19
FVC (l)	2.97 ± 0.89
FVC (z-score)	0.34 ± 1.24
**Genotype, n (%)**	
F508del homozygous	10 (41.7)
F508del heterozygous	14 (58.3)
**Clinical diagnoses, n (%)**	
Exocrine pancreatic insufficiency	22 (91.7)
CF-related diabetes mellitus	1 (4.2)
Liver disease	10 (41.7)
**Microbiology, n (%)**	
Chronic Pseudomonas aeruginosa	4 (16.7)
Chronic methicillin-resistant	0 (0.0)
Chronic Burkholderia cepacia	0 (0.0)

Note: Values expressed as mean ± SD, standard deviation, and with n, number, and %, percentage. Abbreviations: CF, cystic fibrosis; FEV1, forced expiratory volume in first second; FVC, forced volume capacity.

**Table 3:** MEP, MIP, and lifestyle in healthy and CF patients.

	HEALTHY (n = 24)	CF (n = 24)	*p*-Value	Cohen’s d
**MEP and MIP**				
MEP max	88.97 ± 17.51	84.92 ± 16.95	0.440	0.225
MIP max	84.88 ± 20.77	93.21 ± 26.56	0.206	−0.371
MEPmax/MIPmax ratio	1.05 ± 0.33	0.911 ± 0.32	0.152	0.423
**Lifestyle**				
KIDMED score	8.67 ± 1.44	7.54 ± 1.84	0.022	0.682
PAQ C	3.39 ± 0.47	2.94 ± 0.52	0.023	0.896
PAQ A	2.69 ± 0.44	2.25 ± 0.60	0.091	0.824
PAQ total	3.12 ± 0.57	2.65 ± 0.64	0.010	0.774

Note: Values expressed as mean ± SD, standard deviation, and with n, number, and %, percentage. Abbreviations: CF, cystic fibrosis; MEP, maximum expiratory pressure; MIP, maximum inspiratory pressure; KIDMED, Mediterranean Diet Quality Index questionnaire; PAQ-A, Physical Activity Questionnaire for Adolescents; PAQ-C, Physical Activity Questionnaire for Children.

## Data Availability

The original contributions presented in this study are included in the article. Further inquiries can be directed to the corresponding authors due to ethical reasons.
